# Spiritual Distress and Spiritual Needs of Chronically Ill Patients in Poland: A Cross-Sectional Study

**DOI:** 10.3390/ijerph19095512

**Published:** 2022-05-01

**Authors:** Maciej Klimasiński, Ewa Baum, Joanna Praczyk, Monika Ziemkiewicz, Daria Springer, Szczepan Cofta, Katarzyna Wieczorowska-Tobis

**Affiliations:** 1 Department of Palliative Medicine, Poznań University of Medical Sciences, Osiedle Rusa 55, 61-245 Poznań, Poland; tobis@ump.edu.pl; 2 Department of Social Sciences and Humanities, Poznań University of Medical Sciences, ul. Rokietnicka 7, 60-806 Poznań, Poland; ebaum@ump.edu.pl; 3 Mother and Child Specialized Medical Center, ul. Wrzoska 1, 60-663 Poznań, Poland; praczyk.joanna@gmail.com; 4 Ludwik Rydygier Integrated Hospital, ul. Św. Józefa 53-59, 87-100 Toruń, Poland; monikaziemkiewicz@wp.pl; 5 Department of Pulmonology, Allergology and Pulmonary Oncology, University Hospital of Lord’s Transfiguration, Poznań University of Medical Sciences, ul. Przybyszewskiego 49, 60-355 Poznań, Poland; daria.springer.pl@gmail.com (D.S.); szczepan.cofta@skpp.edu.pl (S.C.)

**Keywords:** chronic illness, spiritual care, spiritual distress, spiritual needs, spiritual wellbeing

## Abstract

Introduction: Spiritual care is needed in a clinical setting to improve the patients’ quality of life. Deep connection with another person and delight with the beauty of nature or art and (in some cases) with God are all transcendental experiences. They may enable patients to ascribe meaning to their life with a chronic illness, find hope and well-being despite burdening symptoms. The opposite situation: lack of inner peace, inability to accept what is happening, feeling disconnected from others is called spiritual distress. Objectives: The aim of this research is to assess spiritual distress and spiritual needs of a group of Polish chronically ill patients and find associations with independent variables in order to provide data for recommendations on spiritual care in Poland. Patients and methods: 204 patients treated at the University Hospital and the Cystic Fibrosis Clinic in Poznan were surveyed in 2017 and 2018 with an original questionnaire. Results: Over half of the patients felt that their illness was life-threatening. A little more than half reported that faith was a resource to cope with suffering. Almost all patients showed signs of spiritual distress, and more than half expressed spiritual needs. The intensity of distress correlated only with the severity of the disease. The most important predictor of having spiritual needs was recognizing faith as a resource. Conclusions: Spiritual needs are associated with personal beliefs; however, spirituality spans beyond the religious context since spiritual distress is unrelated to the level of religious devotion. Therefore, any patient with a severe chronic disease needs basic spiritual care, which includes being treated with compassion.

## 1. Introduction

### 1.1. Background and Aims of the Study

Chronic illness, apart from physical ailments, causes considerable suffering that challenges the psychological, social and spiritual dimensions of a patient’s life [1,2,3]. Personal beliefs may affect quality of life by giving structure to an experience, ascribing meaning to it, being a source of comfort, well-being, security and sense of belonging [4]. Spiritual care is an aspect of holistic medicine recognized by modern healthcare systems. Polish patients’ rights law requires all hospitals to employ chaplains. They carry out religious services and the sacraments are always available for those who ask for them [5].

However, there are not any Polish recommendations on how exactly the rest of the medical staff can incorporate spiritual care into everyday clinical practice and which patients need it the most. Therefore, the aim of this study is to assess a group of Polish chronically ill patients and answer the following questions: Do they express signs of spiritual distress? Do they express spiritual needs? What are the characteristics of patients who have stronger distress/needs than others?

### 1.2. Literature Review

Spiritual care may be understood as identifying spiritual needs of the patients and addressing them in a clinical context. It is not limited to the provision of religious resources because a deep connection with another person and delight with the beauty of nature or art are also considered to be spiritual experiences [2,3]. Spirituality may be a resource to cope with the long-term burden of a chronic disease [6]. Spiritual experiences may give patients the opportunity to reevaluate their lives and appreciate their reality despite tiredness and pain [6,7,8,9,10]. Another term used in this field is “spiritual distress”, which is understood as a lack of inner peace and connectedness to loved ones, inability to accept what is happening, find meaning in life and hope for the future. Spiritual distress is the opposite of spiritual wellbeing [11,12,13]. Spiritually distressed patients appear sad, desperate, scared, anxious or angry. They may talk about loneliness, emptiness, uselessness, guilt, injustice, meaninglessness, helplessness [14,15,16]. Some research papers on spiritual distress of seriously ill patients looked into the abovementioned signs of distress rather than asking directly about it. Gielen et al. [17] found the following statements to be true for adult cancer patients: “I feel lonely” (57.7%); “I am afraid of the future” (55.5%); “I am angry because of what is happening to me” (55.3%); “I find it difficult to forgive myself for the wrong I did” (52%); “My life has no purpose” (41.7%).

There is a multitude of publications that list and quantify spiritual needs of adult patients in great detail. Bussing et al. [18], Offenbaecher et al. [19] and Hocker et al. [20] surveyed people with chronic illnesses and cancer. They reported that many of them were looking for “inner peace”, needed to share their fears and concerns with others and “talk to someone about the meaning of life”. Davison et al. [21] surveyed people with chronic kidney failure, whereas Moadel et al. [8], Astrow et al. [22] and Winkelman et al. [23] looked at oncological patients at various stages of cancer. Some of them declared that they needed help to define the meaning of their lives. Pearce et al. [24] also interviewed people with cancer. Some of them said that during their hospital stay, they wanted to make sense of what had happened to them. Thoughts about the meaning of life and suffering lead some people towards faith and religion. In available research, some patients said that they attended church services [20,24,25] or expressed the need for spiritual resources [8,19,21,22,24] or prayer [18,19,20,23,24,25]. Koenig [26], Bussing et al. [27] and Kumar et al. [28] carried out questionnaires among patients with chronic conditions of various cultures. Some of them admitted that spirituality helped them cope with suffering.

The study authors explored published research conducted on patients with chronic illness for predictors of expressing spiritual distress and spiritual needs. In the majority of them, no association with medical characteristics was observed. Bussing [29] stated that religious needs can be predicted best by patients’ intrinsic religiosity (being connected with and guided by a higher source). Yong et al. [30], who surveyed Korean patients with cancer, also reported that the intensity of spiritual needs varied greatly depending on religious beliefs (people without religious denomination demonstrating a lower intensity of needs). Baum [31] found that in a group of patients undergoing hemodialysis, the importance of religion in coping with illness correlated positively with a more significant meaning of life, whilst the sense of a reduced meaning of life correlated with increased severity of symptoms.

These findings suggest that some patients need more spiritual care than others. It is interesting to see if spiritual distress and spiritual needs are associated with severity of the disease or with faith. Most of the research studies up to date have been conducted in the USA (religious society) and in Western Europe (more secularized societies). Poland is unusual with reference to the fact that about 90% of Poles identify with the Catholic Church. However, a considerable number of them may be described as lapsed Catholics (they have faith but do not actually attend any religious meetings).

## 2. Methods

### 2.1. Study Group

Permission for the study was granted by the Head of the University Hospital of Lord’s Transfiguration in Poznan. The inclusion criteria comprised of: hospitalization in one of the hospital departments (Pulmonology, Allergology, Oncology and Cardiology, Hypertensiology and Metabolic Diseases) or treatment at the Cystic Fibrosis Outpatient Clinic and informed consent obtained from the participants. In total, 255 patients were handed the survey between the years 2017 and 2018. Everyone was informed about the purpose of the study and confidentiality was guaranteed. Older patients of the departments, who had difficulty completing the questionnaire, were assisted by an interviewer.

### 2.2. Study Measures

In order to quantify both signs of spiritual distress and spiritual needs of the study group, a proper questionnaire was needed. The research cited above [8,18,19,20,21,22,23,24,25,26,27,28] provides many tools, for example, the SpREUK-15, SNI, FACIT-Sp and SpNQ, which have been validated in Poland [29]. There are also numerous spiritual distress questionnaires that assess up to 40 clinical indicators [32]. Recently, measures used in screening for spiritual experiences have been reviewed by Bahraini et al. [33] and Damberg Nissen et al. [34]. While these tools are of great value, none of them were chosen, because we preferred to use a simple, short, questionnaire developed ad hoc. This is a very similar approach to Davison et al. [21], Astrow et al. [22] and Winkelman et al. [23] who also asked questions about spiritual needs that were not considered to be a newly developed tool. The statements provided by us have some overlap with items in existing validated tools, e.g., our items A5; A8; A9; A10; A11; A12 are comparable to SpNQ items 4, 27; 11; 10; 2, 13; 22; 20, 23, respectively, and our item A8 is also comparable to FACIT-Sp-12 items 2, 5, 8.

Part A of the newly developed questionnaire provided answers A1–A12 in the five-point Likert’s scale. Items A1–A10 looked into the signs of spiritual distress and items A9, A11 and A12 into spiritual needs. Part M assessed a number of independent variables that would later be used to be correlated with the intensity of expressed spiritual distress and spiritual needs. It contained open questions about diagnosis, age and sex (M1 to M3), a question to subjectively determine the severity of illness (M4, Likert’s scale) and three questions about faith and its importance in one’s life: Which religious denomination do you belong to (M5, multiple choice), are you a member of a religious group (M6, dichotomous), do you perceive faith as a resource to cope with your condition (M7, Likert’s scale). In all questions containing Likert’s scale, the frequency of occurrence of a given phenomenon was calculated by adding the frequencies of “definitely yes” and “rather yes” answers. The study protocol was accepted by the Independent Ethics Committee at Poznan University of Medical Sciences (Resolution number 709, of 16 June 2016). The originally developed questionnaire was then piloted among 30 chronically ill volunteers through a test–retest setup. It was submitted to them twice, with an interval of 2 to 4 weeks. This amount of time is long enough so that the respondent would not remember the details of his/her previous answers, but also not too long so that no major life events should happen, because that would affect the patient’s responses. The analysis of this validation is presented in the Results section.

### 2.3. Statistical Analyses

Statistical analyses were performed using IBM SPSS Statistics 25 (IBM Corp. 2017. IBM SPSS Statistics for Windows. Armonk, NY, USA). The Shapiro-Wilk tests were carried out with a number of *r* Pearson’s and Spearman’s rho’s correlation analyses, as well as Student’s *t* tests for independent samples along with Mann–Whitney’s tests. The alpha = 0.05 was assumed as the level of significance. In addition, principal components analysis (PCA) with VARIMAX rotation as well as multivariate linear regression were performed for quantitative, dependent variables.

## 3. Results

### 3.1. Validation Results

The reliability was assessed by Cronbach’s alpha coefficient. It was high for “Signs of Spiritual Distress” both before and after the interval (0.873 and 0.837, respectively). It was also high for “Spiritual needs” both before and after the interval (0.848 and 0.785, respectively). Its repeatability was analyzed with Cohen’s kappa significance test for every single item separately, it ranged between 0.570 and 0.857, which represents moderate to perfect repeatability. Therefore, the questionnaire proved to be an effective questionnaire for measuring the studied phenomena.

### 3.2. Demographic Characteristics and Other Independent Variables (Part M of the Questionnaire)

A group of 204 patients completed the survey (80% response rate). Of all respondents, 61.4% were women and 38.6% were men. The average age was 52.9 years (min: 18 years, max: 94 years old). The surveyed patients were diagnosed with: cystic fibrosis (52 people—25.4%), followed by other lung disease (24 people—11.6%), primary heart disease (23 people—11.1%), cancer (23 people—11.1%), chronic obstructive pulmonary disease (21 people—10.6%), hypertension (21 people—10.1%), systemic disease (12 people—5.3%), asthma (9 people—4.2%), diabetes (7 people—3.7%), pneumonia (7 people—3.7%) and sleep apnea (6 people—3.2%). Over half of the surveyed acknowledged that their illness was life-threatening (59.6%). Almost all respondents (89.5%) described themselves as Catholics, 6.5% as atheists and 4.0% as people of another religion. A little more than half (62%) reported that they did not actually participate in any religious group. A little over half of all patients (54.7%) reported that faith was a resource to cope with the burden of long illness.

### 3.3. Frequency, Reliability of Items, Factor Analysis of the Part A of the Questionnaire

Table 1 presents the answer to the first and second study question: the frequency of reported signs of spiritual distress and spiritual needs, along with an analysis of their reliability. In order to check whether the theoretical factor structure is consistent with the empirical structure, factor analysis was performed using the principal component method (PCA). In the first step, the exploratory method examined how many dimensions can be extracted from the scree plot and initial eigenvalues greater than 1 (Table 2). The KMO result was 0.82, which indicates that the analysis is justified. The obtained results indicate that, according to the assumptions, two factors can be distinguished, accounting for over 52% of variance (Table 2). In the next step, an analogous analysis was performed, but with an exploratory method (a two-factor solution was imposed on the algorithm) along with VARIMAX rotation (Table 2). Actually, according to the assumptions, the first factor includes items A1, A2, A3, A4, A5, A6, A7, A8 and A10. The last item, however, heavily loads both factors similarly.

### 3.4. Correlation Analysis and Multivariate Linear Regression of Part A of the Questionnaire and Independent Variables (Part M of the Questionnaire)

Correlation analysis and multivariate linear regression were performed to answer the third study question: “What are the characteristics of patients who have stronger distress/needs than others?”. Firstly, the distribution of quantitative variables was checked. For this purpose, the basic descriptive statistics and the Shapiro-Wilk test were performed (Table 3). Excessive kurtosis was used as a measure of kurtosis. Table 4 presents the calculated correlation coefficients of quantitative independent variables (age, faith being a resource in illness, severity of illness) and the results of Student’s *t* tests for dichotomous independent variables (gender, being a member of religious group). Statistically significant are the following connections between: (1) The intensity of “Signs of Spiritual Distress” and the severity of the disease; (2) “Spiritual Needs” with: age, gender, participation in a religious group and acknowledging faith as a resource in illness. In order to deepen the analysis, a multivariate linear regression was conducted for “Spiritual Needs”. The positive coefficients of the analysis showed that the most important predictor was faith as a resource in illness. The weaker predictors were: age and participation in a religious group. Gender did not turn out to be an independent predictor (Table 5).

## 4. Discussion

### 4.1. Summary of Findings and Interpretation of Results

More than a half of the respondents showed signs of spiritual distress (stress, sadness, fear of what is to come, the need to share thoughts and feelings with others), as well as a spiritual need to pray. Correlation analysis and multivariate linear regression allow for two important observations. Firstly, the fact that spiritual distress is associated with severity of the disease may confirm the assumption that spirituality spans beyond the religious context. It seems that regardless of the level of religious devotion, a high symptom burden is difficult to accept. It can disrupt inner peace, relationships with loved ones, strain the meaning of life and blur the hope for the future. Secondly, the fact that in this study spiritual needs are associated with acknowledging faith as a resource to cope with illness points to the possibility of predicting which patients have stronger spiritual needs than others. It should be made clear, however, that these assertions are based on findings from an ad hoc questionnaire that is not fully theoretically grounded and not entirely congruent with conceptual or terminological definitions by other scholars.

### 4.2. Methodological Considerations

Firstly, the questionnaire used in this study consisted of items concerning both spiritual distress and spiritual needs, picked by the authors. There was no development phase, no interviews to conceptualize items (only literature review) and the survey was validated only in a test–retest manner on a group of 30 participants. In future research in Poland, broadly used, well-developed (e.g., without items that contain two questions in one, like our A1 item) and well-validated questionnaires should be used.

The signs of spiritual distress that the authors have chosen to measure may be perceived as signs of depression, grief or other emotional states. Therefore, one could argue that this is not an area of interest of spiritual care but of clinical psychology instead. Clinical psychology deals with the patient’s psyche, emotional balance, anxiety disorders and addictions. A psychologist working in a hospital is called for a consultation when a sudden event occurs that causes a severe emotional reaction in the patient (i.e., burdening diagnosis, life-threatening condition of a loved one, etc.). Such a specialist is an excellent listener, he/she can name and react to patients’ emotions and have a supportive conversation with them. However, chronically ill patients may not present acute emotional reactions. If the illness has caused them to lose the meaning of their lives and hope for the future, they may feel sad, lonely, distrustful for months or even years. Usually, an attending physician would not call a clinical psychologist for an intervention, since there is not really any urgent matter that could be resolved.

Secondly, when the distribution of quantitative variables was checked by calculating the basic descriptive statistics the results indicated that the distributions of all variables were statistically different from the normal distribution. The skewness, however, was in the correct range, which means that further calculations are valid.

### 4.3. Other Limitations

Taking into account the fact that it is a cross-sectional study, a few important limitations are outlined here. Firstly, the patient groups that were compared with each other are possibly very heterogeneous. Other coefficients, such as: stage of the disease, possibility for a definite cure, etc., could have affected the outcome of correlation analysis.

The second limitation is the fact that we did not determine whether the intensity of signs of spiritual distress and intensity of spiritual needs change over time (before the illness, after diagnosis, a few years later). Consequently, it is not possible to distinguish causes from effects. This applies mainly to the correlation analysis of dependent variables. Worsening symptoms of a chronic illness will most probably cause patients to become more spiritually distressed. However, it could be argued that something else had caused the spiritual distress in the first place, and the distress led to worsening of symptoms (due to worse compliance, worse immunological function, etc.). Another example: does being a member of a religious group intensify patients’ spiritual needs? It could be the other way round: the intensity of these needs could make the person engage in religious practices. The third limitation is the lack of an analysis of whether any change in perceiving faith as a resource in illness and undertaking religious practices occurred after diagnosis.

## 5. Conclusions

This study is among the first ones to look at the spiritual distress and spiritual needs of Polish patients. We found that signs of spiritual distress (such as lack of inner peace, sadness, anger, fear of what is to come) were indeed common for our group of 204 participants with chronic conditions, and around half of them also expressed the need to pray. Our findings suggest that it is likely that patients for whom faith is a resource will benefit the most from facilitation of those needs in a medical setting. Some of these patients may be unwilling to spontaneously express such needs, so we recommend using a screening question at admission. In the past, most Polish patients were Roman Catholics, and it was assumed they would welcome a priest. This may not be the case anymore. The change of the way that chaplains visit hospital wards is definitely an option worth considering in the future.

In the present study spiritual distress was independent of faith being a resource in illness and of being a member of a religious group. Its intensity increased only with the severity of the disease. Properly trained members of medical staff can provide support to patients who do not identify with any particular religion. In fact, they can accompany anyone who feels sad, lonely, restless or irrelevant. Therefore, any member of the hospital staff is an appropriate candidate to pay extra attention and show compassion to such patients, perhaps encourage them with a kind word or a delicate touch. However, to name these actions as provisions of the basic spiritual care is not yet common in Poland. Similarly, doctors and nurses are rarely seen as members of the spiritual care team. It is time to make the terms spiritual distress, spiritual needs and spiritual care known more broadly in Poland. Proper education about spiritual needs, spiritual wellbeing and spiritual distress in Polish medical schools is needed to improve the quality of care in Polish hospitals. Certainly, more research is needed to determine how exactly spiritual care should be provided by Polish medical personnel. Hopefully, this study will inspire research on practical applications of spiritual care in Poland.

## Figures and Tables

**Table 1:** Spiritual experiences related to illness.

	Prevalence (% of Patients)	Cronbach’s Alpha
“Signs of Spiritual Distress”		0.839
A1. I feel stressed, lack of peace of mind	61%	0.820
A2. I think that nobody understands me	31%	0.820
A3. I feel sad	55%	0.828
A4. I feel anger	44%	0.822
A5. I think that I could have lived my life better	31.5%	0.836
A6. I fear of what is to come	64%	0.817
A7. I think that my plans for the future are pointless	25%	0.816
A8. I think that my life lacks meaning and purpose	14%	0.815
A10. I need to share thoughts and feelings with others	51.5%	0.829
“Spiritual Needs”		0.737
A9. I need to find meaning in suffering	27%	0.833
A11. I need spiritual resources	37.7%	0.535
A12. I need to pray	51.5%	0.509

**Table 2:** Factor analysis.

Total Explained Variance for the Exploratory Method of Principal Components Analysis							The Results of Two-Factor Solution after Rotation		
Component	Initial Eigenvalues			Sum of Squares of Charges after Extraction			Item	Matrix of Rotated Components	
	Total	% Variance	% Cumulative	Total	% Variance	% Cumulative		First Factor	Second Factor
1	4.593	38.275	38.275	4.593	38.275	38.275	A1	0.630	
2	1.672	13.929	52.204	1.672	13.929	52.204	A2	0.649	
3	0.907	7.560	59.764				A3	0.597	
4	0.798	6.647	66.411				A4	0.717	
5	0.729	6.077	72.488				A5	0.509	
6	0.681	5.675	78.162				A6	0.676	
7	0.613	5.111	83.274				A7	0.748	
8	0.555	4.623	87.896				A8	0.755	
9	0.542	4.514	92.410				A9	0.458	0.496
10	0.385	3.206	95.616				A10	0.436	0.555
11	0.303	2.526	98.142				A11		0.888
12	0.223	1.858	100.000				A12		0.886

**Table 3:** Descriptive statistics with testing of the normality of distribution.

	M	Mdn	SD	Sk.	Kurt.	Min.	Max.	S-W	*p*
Age	52.91	58.00	19.70	−0.24	−1.09	18.00	94.00	0.95	<0.001
Is the illness life-threatening?	3.60	4.00	1.16	−0.42	−0.92	1.00	5.00	0.88	<0.001
Is faith a resource in illness?	3.35	4.00	1.32	−0.49	−0.71	0.00	5.00	0.90	<0.001
“Signs of Spiritual Distress”	2.92	2.94	0.88	0.18	−0.67	1.00	5.00	0.98	0.009
“Spiritual Needs”	2.98	3.00	1.06	−0.02	−0.82	1.00	5.00	0.97	<0.001

**Table 4:** Correlation analysis between dependent and independent variables.

	Age	Gender	Life-Threatening Illness	Member of Religious Group	Faith is a Resource in Illness
“Signs of Spiritual Distress”	Pearson’s *r* −0.13	Student’s *t* 0.89	Pearson’s *r* 0.33	Student’s *t* 1.11	Pearson’s *r* 0.07
	*p* 0.071	*p* 0.377	*p* < 0.001	*p* 0.271	*p* 0.331
“Spiritual Needs”	Pearson’s *r* 0.26	Student’s *t* 2.44	Pearson’s *r* 0.05	Student’s *t* 3.61	Pearson’s *r* 0.49
	*p* < 0.001	*p* 0.016	*p* 0.449	*p* < 0.001	*p* < 0.001

**Table 5:** Multivariate linear regression; “enter” method.

Dependent Variable: “Spiritual Needs Index” Adjusted R^2^ = 0.335				
Predictors	B	Standard Error	Beta	*p*
Faith is a resource in illness	0.358	0.055	0.428	<0.001
Age	0.012	0.003	0.222	0.001
Member of religious group	0.388	0.140	0.177	0.006
Gender	−0.192	0.138	−0.088	0.166

## Data Availability

Data supporting reported results cannot be found in any publicly archived datasets. The data can be provided upon request.
